# Case report; successful treatment of traumatic ischaemic hemiplegia secondary to blunt carotid injury associating high grade liver trauma

**DOI:** 10.1016/j.ijscr.2021.106547

**Published:** 2021-11-02

**Authors:** Ayman O. Nasr, Turki Muslih Al-Harbi, Fatimah Sami AlRamadan

**Affiliations:** a Department of Surgery, King Fahad Hospital Of University-Collage of Medicine, Imam Abdulrahman Bin Faisal University, Saudi Arabia; b Collage of Medicine, Imam Abdulrahman Bin Faisal University, Saudi Arabia

**Keywords:** Case report, Blunt carotid injury, Traumatic hemiplegia, Liver injury

## Abstract

**Introduction and importance:**

Blunt carotid injury (BCI) injury is a rare sequel of trauma and could result in ischemic complication if not detected and treated early. The presence of high-grade solid organ injury with ongoing bleeding represents additional challenge in treating BCI.

**Case presentation:**

A 25-year-old victim of motor vehicle collision resulted in grade IV liver, grade III left kidney and grade I spleen injury. He underwent an urgent laparotomy with transient liver packing at local hospital. A full body Contrast-Enhanced Computer Tomography (CECT) upon arrival revealed right internal carotid intimal tear with intra and extra-cranial thrombosis and a 3 cm aneurysm. With a decreased level of consciousness, the patient showed a GCS of 13 and left-sided hemiplegia. After complex multidisciplinary treatment sessions, patient recovered with a partial regain of left-sided muscle power.

**Clinical discussion:**

Selective embolization of active liver bleeding was a turning point in the management of our patient as it deferred the need for a second operative intervention. It was a necessary step before endovascular stenting and recanalization of the BCI to restore the circulation to the right cerebral hemisphere. Dual anti-platelet therapy (DAPT) was necessary to prevent thrombosis of the stent and continuity of carotid recanalization.

**Conclusion:**

BCI with traumatic ischaemic hemiplegia associating a sum of life-threatening multiple injuries including high grade liver trauma with ongoing bleeding could still be managed non-operatively with acceptable outcome in the presence of a comprehensive specialized multidisciplinary service.

## Highlights

• Early detection and high susception of blunted carotid injury (BCI) prevent deterioration and promote better prognosis.
• BCI associated with polytraumatic injuries can be managed non-operatively with acceptable outcome.
• The use of dual antiplatelet therapy in anticoagulant contraindicated patients is essential to prevent stent-related thrombosis.

## 1 Introduction

Blunt carotid injury (BCI) is reported in 1–2.6% of blunt trauma cases [1]. Untreated extra-cranial carotid artery injuries are associated with a 60% rate of ischemic stroke and up to 43% mortality which could be significantly reduced if timely treated [2]. The rare incidence of BCI as a result of road traffic accidents accompanies other organ damage including liver trauma that will eventually change the treatment plan [3].

The treatment protocol of high-grade liver trauma has changed considerably from a surgical intervention towards a more non-surgical approach [4]. Advancement in interventional radiology allowed selective embolization of active arterial bleeding deferring the need for peri-hepatic packing [5] and damage control surgery to haemodynamically unstable patients [6].

We report a case of a complicated BCI associating multiple high-grade solid organ injuries requiring multi-stage surgical and interventional radiological interventions. The work has been reported in line with the SCARE criteria [7].

## 2 Case report

A 25-year-old male patient, a victim of a motor vehicle collision, was brought to the emergency department of the nearest medical facility which was a private hospital due to haemodynamic instability. The patient underwent immediate laparotomy which revealed 2 l of intra-peritoneal free blood, deep liver laceration involving segments IV_a & b_, V, VI and VIII, longitudinal mesenteric laceration and retroperitoneal haematoma at zone-II. Liver packing for 15 min was performed with approximation of mesenteric defect and insertion of two large abdominal drains. The abdomen was closed and the patient was admitted to the intensive care unit.

**Fig. 1:**
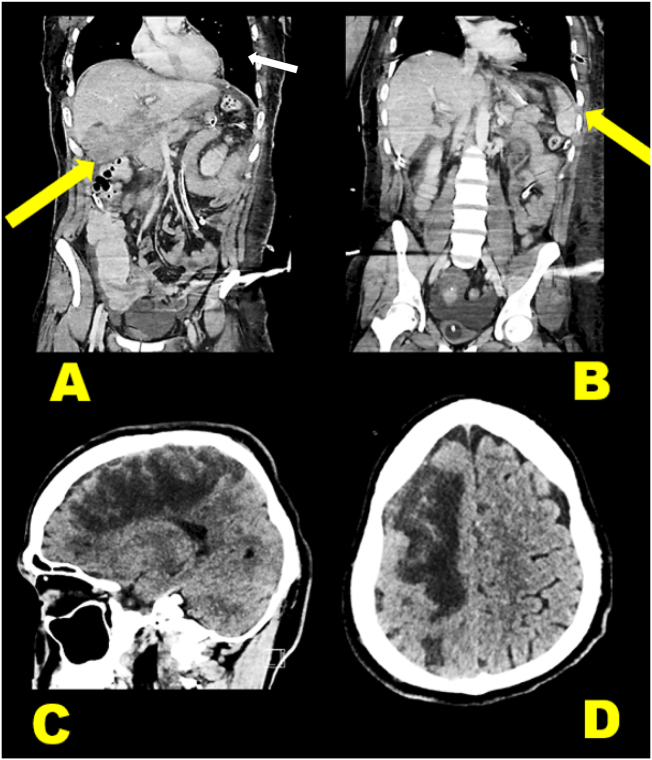
Major CT findings, A; Grade-II splenic injury (arrow), B; Grade-IV Liver injury (arrow), C & D; Rt. Fronto-parietal cerebral haematoma.

The patient was transferred to our facility ventilated, in a stable condition, he underwent full body Contrast-Enhanced Computer Tomography (CECT) (Fig. 1) upon arrival which revealed right fronto-parietal hematoma, bilateral pneumothorax with left-sided haemothorax, grade-IV liver injury [8] with evidence of active bleeding, grade-I spleen laceration, grade-III left kidney, T12, L1–3 left transverse processes fractures and retroperitoneal haematoma (Fig. 3). CECT also revealed right internal carotid intimal tear with intra and extra-cranial thrombosis and a 3 cm aneurysm. With decrease level of consciousness, the patient showed a GCS of 13 and left-sided hemiplegia.

**Fig. 2:**
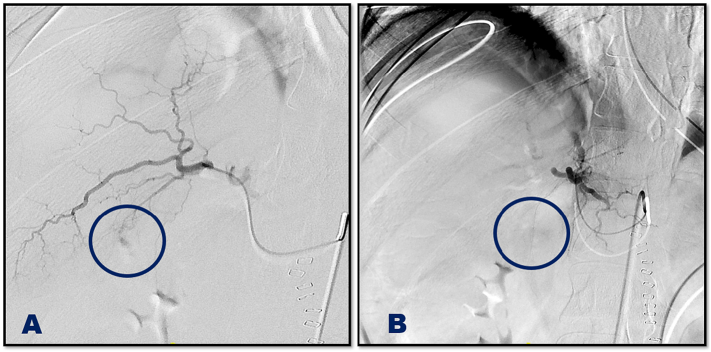
Selective embolization of Liver injury, A; before embolization, B; after embolization.

**Fig. 3:**
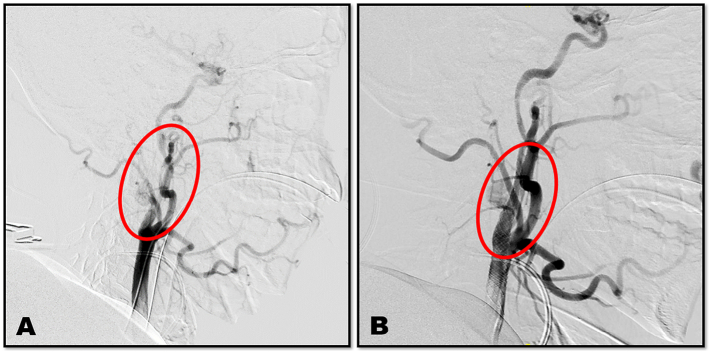
Rt. Carotid artery stenting, A; before stenting, B; after stenting.

As the patient was haemodynamically stable, selective interventional embolization of the active liver bleeding (Fig. 2) followed by right carotid double-coated stent insertion and thrombolysis was performed by the interventional radiology unit (Fig. 3). The patient required dual anti-platelet therapy (DAPT) (aspirin and clopidogrel) to prevent stent-related thrombosis and improve the outcome of the concomitant ischaemic hemiplegia. This represented a management challenge in the presence of multiple sold organ injuries and retroperitoneal and intracranial haematomas that required close monitoring and high preparedness to intervene.

The patient stayed in ICU for three weeks before being discharged to the regular ward with very critical times of increase in haematoma size. With appropriate physiotherapy, the patient started after one month to move the lower limbs (grade-II power) and evidence of complete healing of intra-abdominal injuries.

## 3 Discussion

Traumatic ischaemic hemiplegia secondary to BCI in the presence of severe multiple solid organ injuries was not reported in literature based on a comprehensive the literature search. Reports of high-grade liver trauma associated with major vascular trauma were only found in relation to aortic trauma [9]. Endovascular stenting of BCI is documented as the treatment of choice with good outcome when identified and managed early [10]. The presence of severe intra-abdominal trauma with haemorrhagic hypotension requiring immediate intervention has resulted in delayed detection of BCI in our patient with consequent ischemic hemiplegia. Hence, an immediate change of the treatment plan was required to improve the outcome of the stroke.

Selective embolization of active liver bleeding was a turning point in the management of our patient as it deferred the need for a second operative intervention. It was a necessary step before endovascular stenting and recanalization of the BCI to restore the circulation to the right cerebral hemisphere. DAPT was necessary to prevent thrombosis of the stent and continuity of carotid recanalization [11]. It is indicated post stenting not only to prevent the ischemic complication of carotid stenting, it also has a beneficial role in a patient with increased risk of ischemic events [12]. The use of antiplatelet therapy for those for whom anticoagulation is deemed contraindicated has been shown to be essentially equivalent by some authors [13].

The presence of interventional angioembolization and neuro-endovascular hybrid unit at our facility supported the non-operative approach on our patient. Lack of comprehensive specialized multidisciplinary service under one roof made the initial damage control laparotomy, a justified approach [14] by the first receiving hospital. The same facility allowed the utilization of endovascular stenting with the associated risk of rebleeding from DAPT [15].

In conclusion, BCI with traumatic ischaemic hemiplegia associating a sum of life-threatening multiple injuries including high grade liver trauma with ongoing bleeding could still be managed non-operatively with acceptable outcome in the presence of a comprehensive specialized multidisciplinary service.

## Funding

The authors deny any source of funding in this project.

## Ethical approval

The institutional review board approved the publication of this case report with a reference number: IRB-2021-01-291.

## Consent

Written informed consent was obtained from the patient for publication of this case report and accompanying images. A copy of the written consent is available for review by the Editor-in-Chief of this journal on request.

## Author's contribution

Ayman Nasr: Treating consultant, main author, writing the manuscript.

Turki Al Harbi: Data collection and writing the manuscript.

Fatimah AlRamadan: Data collection and writing the manuscript.

## Registration of research studies

N/A.

## Guarantor

Dr. Ayman Nasr.

## Provenance and peer review

Not commissioned, externally peer-reviewed.

## Declaration of competing interest

The authors declare that there is no conflict of interest regarding the publication of this paper.
